# Long-term retention of the pedicled thymic flap after bronchial stump coverage

**DOI:** 10.1093/icvts/ivaf012

**Published:** 2025-01-28

**Authors:** Takahiro Karasaki, Sakashi Fujimori, Souichiro Suzuki, Shinichiro Kikunaga, Kazuki Ito, Yosuke Hamada, Shusei Mihara

**Affiliations:** Department of Thoracic Surgery, Respiratory Center, Toranomon Hospital, Tokyo, Japan; Department of Thoracic Surgery, Respiratory Center, Toranomon Hospital, Tokyo, Japan; Department of Thoracic Surgery, Respiratory Center, Toranomon Hospital, Tokyo, Japan; Department of Thoracic Surgery, Respiratory Center, Toranomon Hospital, Tokyo, Japan; Department of Thoracic Surgery, Respiratory Center, Toranomon Hospital, Tokyo, Japan; Department of Thoracic Surgery, Respiratory Center, Toranomon Hospital, Tokyo, Japan; Department of Thoracic Surgery, Respiratory Center, Toranomon Hospital, Tokyo, Japan

**Keywords:** minimally invasive surgery, video-assisted thoracoscopic surgery, complications

## Abstract

This study aimed to assess the feasibility and retention rates of pedicled thymic flaps to enhance understanding of bronchial stump coverage. A retrospective analysis of 22 consecutive patients who underwent anatomical lung resection followed by bronchial stump coverage with pedicled thymic flaps between January 2017 and December 2022 was conducted. The procedure was performed using a minimally invasive three-port video-assisted thoracoscopic surgery technique for all patients with no complications related to graft harvest or fixation. Postoperative retention of the engrafted flaps was evaluated in the 16 patients who underwent thin-slice computed tomography at least once after surgery. The majority of the postoperative computed tomography examinations were performed for surveillance of lung cancer recurrence. The retention rates of pedicled thymic flaps were 100% at 60 days, 87% at 180 days and 70% at 1 year post-surgery. Pedicled thymic flaps can be safely harvested using a minimally invasive approach, and the majority of engrafted flaps remain adjacent to the bronchial stump for more than 1 year. This technique may be a viable option for patients at high risk of a delayed bronchopulmonary fistula.

## INTRODUCTION

A bronchopulmonary fistula (BPF) is one of the postoperative complications associated with increased surgical mortality following lung resection. The reported incidence of BPF after lung resection has decreased from around 2% to <1% over the past several decades [1–5].

However, with the improvement in perioperative treatment, more patients are expected to undergo pre-operative or postoperative chemo(radio)therapy and immunotherapy, and the risk of delayed healing of the bronchial stump or anastomosis may increase. Of note, a series of BPF cases after neoadjuvant immunochemotherapy has been recently reported [6]. In addition, the upcoming ninth edition of the TNM classification of lung cancer introduces new N2 subcategories, possibly increasing the number of clinically N2 patients eligible for radical resection [7]. This may also increase the number of residual diseases at the bronchial stump or mediastinum, a known risk factor for BPF [8]. Revisiting the risks of BPF and gaining a deeper understanding of bronchial stump coverage methods could therefore improve patient care.

To prevent fatal complications after BPF, bronchial stump coverage and reinforcement using various tissues, including pedicled intercostal muscle, thymus or pericardial fat, have been reported to date [2, 5, 9]. We recently reported that the retention rates of free pericardial fat grafts after bronchial stump coverage were 100% at 60 days after surgery, 61% at 180 days and 25% at 360 days [10]. However, the retention rate of engrafted pedicled thymic flaps remains unknown. This study aimed to investigate the feasibility and retention rate of pedicled thymic flaps to enhance our understanding of bronchial stump coverage and ultimately improve patient care.

## MATERIALS AND METHODS

The Institutional Review Board (No. 2545) approved the study on 11 March 2024. Due to its retrospective nature, written informed consent was waived.

The surgical records and patient charts of individuals who underwent surgery between January 2017 and December 2022 were retrospectively reviewed. The study included consecutive patients who had anatomical lung resection followed by bronchial stump coverage with a pedicled thymic flap.

The criteria for bronchial stump coverage in our institution included, but were not limited to, a history of poorly controlled diabetes mellitus, systemic steroid therapy, lung infection, or prior mediastinal radiotherapy [10]. In cases in which the pedicled thymic flap length was insufficient to reach the lower bronchus, free pericardial fat grafts were used instead. Thus, all patients who underwent bronchial stump coverage with a pedicled thymic flap had diseases affecting the upper or middle lobes. The pedicled thymic flap was primarily harvested using electric cautery and an ultrasound scalpel, and it was secured to the stump and the surrounding tissues with 4–6 stitches of 4–0 polydioxanone sutures. Example photos of the pedicled thymic flap and the bronchial stump coverage are shown in Supplementary Material, Fig. S1.

As previously described [10], physical retention of sufficient engrafted tissue was diagnosed based on the following criteria observed in a thin-slice computed tomography (CT) scan with a slice thickness of 2 mm or less: (i) identification of soft tissue adjacent to the bronchial stump; (ii) the soft tissue had a thickness of 3 mm or more; and (iii) it covered more than 80% of the length of the staple line at the bronchial stump. For example, to meet this criterion, more than 12 mm of the 15 mm length of the bronchial stump should be covered with the graft. If any of these criteria were not met, the engrafted tissue was considered regressed. The retention rate on a certain day after surgery was calculated as follows:

*a* = The number of cases diagnosed with sufficiently retained engrafted tissue identified on CT scan performed after postoperative day (POD) *X*

*b* = The number of cases diagnosed with regressed engrafted tissue on CT scan performed before POD *X*

Retention rate on POD *X* = *a*/(*a* + *b*)

Data analysis and visualization were conducted using R 4.3.3 (R Foundation for Statistical Computing, Vienna, Austria). Confidence intervals of retention rates were estimated using the bootstrap percentile method with 1000 bootstrap samples using the boot (v1.3–30) R package.

## RESULTS

### Patients’ characteristics

During the study period, 22 consecutive patients underwent lung resection followed by bronchial stump coverage using pedicled thymic flaps. The procedure was performed using a three-port video-assisted thoracoscopic surgery technique for all patients. Table 1 provides a summary of the patients’ characteristics. The cohort included 8 female and 14 male patients. Lung resection was performed for tumours in 15 patients and for infectious and/or inflammatory diseases in 7 patients. The procedure was performed on the right side in 19 patients and on the left side in 3 patients. The extent of lung resection included lobectomy in 11 patients, concomitant lobectomy and segmentectomy in 1 patient and bilobectomy in 10 patients. Seven cases developed minor postoperative complications (Clavien-Dindo grade ≤II): 2 patients with a grade II prolonged air leak for 7 days or more that was treated by pleurodesis using the chest tube that was inserted during the surgery and 5 patients with a grade I prolonged air leak that required no additional treatment. One of the patients with a prolonged air leak also developed grade II pneumonia that was treated with antibiotics. None of the patients in the cohort developed major complications that were defined as Clavien-Dindo grade ≥III including BPF during follow-up.

**Table 1:** Patients’ characteristics (*N* = 22)

Variables		
Age (years)		59 (29–77)
Sex	Female	8
	Male	14
Diagnosis	Benign tumour	1
	Malignant tumour	14
	Inflammation or infection	7
Side	Left	3 (13.6)
	Right	19 (86.4)
Surgery type	Upper lobectomy	11
	Upper lobectomy and apical segmentectomy of lower lobe	1
	Upper and middle bilobectomy	1
	Middle and lower bilobectomy	9
Chest tube duration (days)		4.5 (1–16)

Continuous variables are reported as median (range) values, and categorical variables are reported as counts.

### Assessment of retained pedicled thymic flaps on postoperative computed tomography

**Figure 1:**
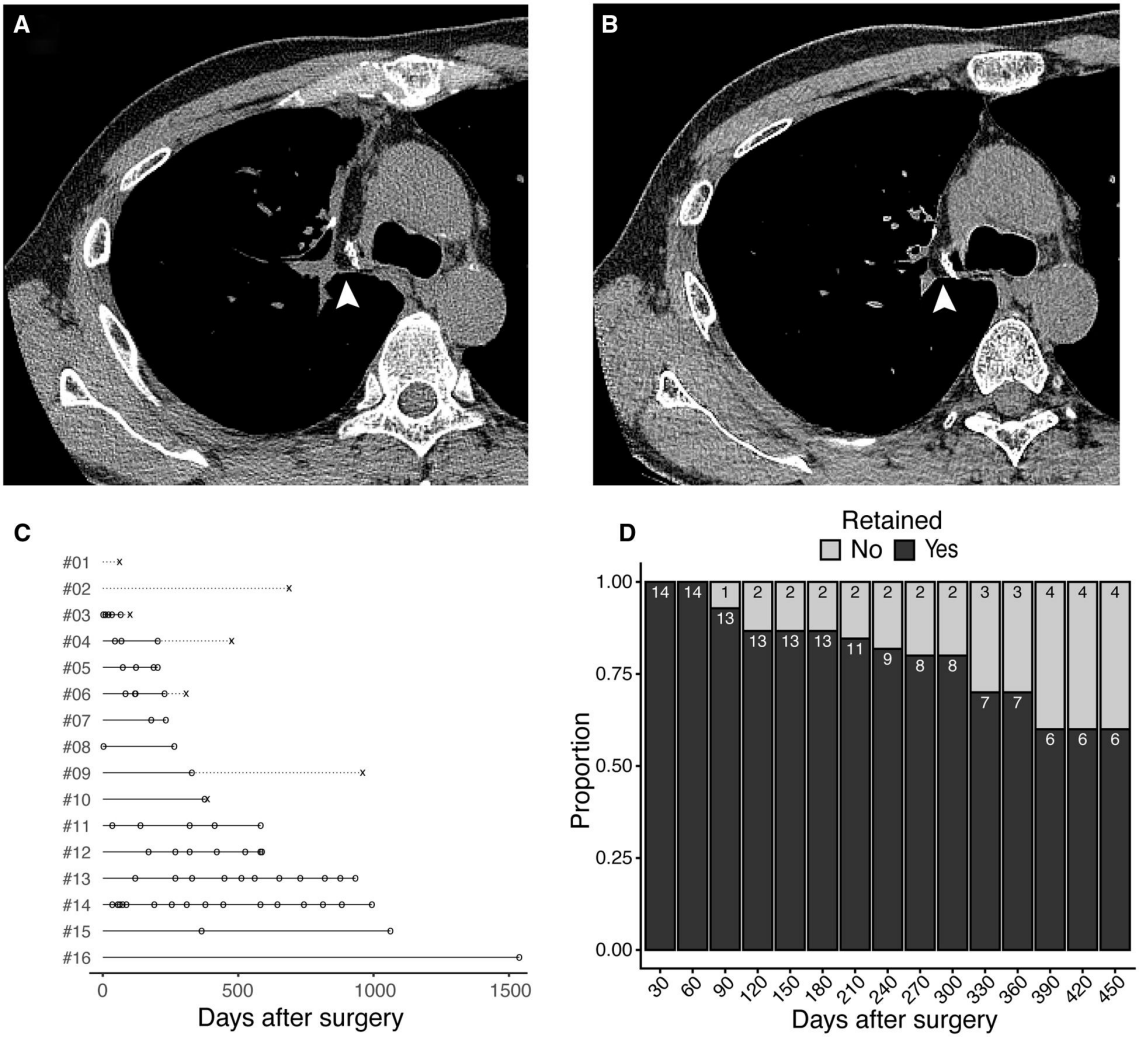
Retention rate of the pedicled thymic flaps after surgery. (**A, B**) Postoperative CT scans showing the retention of the flap adjacent to the bronchial stump (arrowheads) at postoperative days 35 (**A**) and 413 (**B**). (**C**) Swimmer plot showing days of the CT scan. A circle (o) represents the day of the CT scan with a retained flap, and a cross (×) represents the day of the earliest CT scan with a regressed flap. Solid lines represent the periods with a high probability of having a retained flap, and dotted lines represent the period during which the flap has possibly regressed. **(D)** Bar plot showing the proportion of the retained and regressed flaps at each time point after surgery

Sixteen patients, including 13 patients with lung cancer, 2 patients with infection and 1 patient with a benign tumour, underwent thin-slice CT at least once after the surgery that could be analysed for the retention of engrafted pedicled thymic flap. The majority of the CT examinations were performed for surveillance of lung cancer recurrence. Since we routinely use plain CT for postoperative surveillance of lung cancer or for the examination of pneumonia, most of the CT examinations performed in the current study did not use contrast enhancement. Examples of postoperative CT scans are shown in Fig. 1A and B. The median days of the latest CT scan with a retained flap adjacent to the bronchial stump, and the earliest CT scan with a regressed flap were 352.5 (range 66–1538) days and 386 (62–960) days, respectively (Fig. 1C, Supplementary Material, Table S1). The observed retention rate of the graft within the cohort was 100% up to POD 60 and gradually decreased over time. Retention rates at PODs 180, 360 and 450 were 86.7% (95% confidence interval 66.7–100%), 70.0% (40.0–100%) and 60.0% (27.3–90.9%), respectively (Fig. 1D). The retention rates were almost identical when the patients with benign diseases were excluded from the analysis (92.3%, 75.0% and 62.5% at POD 180, 360 and 450, respectively) (Supplementary Material, Fig. S2). All three patients who had regression of the flap within 1 year had detectable engrafted tissue adjacent to the bronchus on postoperative CT, but it did not fulfil the criteria for retention and was therefore considered graft regression.

## DISCUSSION

The forthcoming ninth edition of the TNM classification for lung cancer, along with recent advancements in perioperative treatments, may allow a subset of clinically N2 patients to become surgical candidates. This may increase the number of cases with R1 resection at the bronchial or mediastinal margin, who would be candidates for postoperative radiation. Extensive mediastinal lymph node dissection itself may also have an increased risk of ischaemia around the bronchial stump. Therefore, there is concern that the risk of delayed BPF may increase in a fraction of patients undergoing surgery. The sufficiently long retention rate of the pedicled thymic flap presented in the current study suggests that this technique may be one of the options suitable for patients with a high risk of delayed BPF.

Empirically, harvesting the free pericardial fat graft is less complicated and less time-consuming than a pedicled thymic flap. However, the retention rate of free fat grafts decreases to <50% after 6 months and to around 20% after 1 year [10]. These data raise concerns that a free fat graft might be vulnerable to the risk of delayed BPF. An intercostal muscle flap is another option frequently used in open thoracotomy; however, it is not suitable for a minimally invasive approach. The type of graft used to cover the bronchial stump should be carefully selected based on the risk, feasibility, anatomy, and surgical approach in each case.

### Limitations

This study had several limitations. First, only physical retention of the flaps could be assessed on CT, not the function and viability of the engrafted tissues. Whether the retained flaps reduce the risk of BPF is an issue that was beyond the scope of the study. Second, the retrospective design and small patient sample size limit the generalizability of the findings, necessitating further validation. In addition, postoperative CT was performed on an *ad hoc* basis rather than at regular intervals, introducing potential bias in the timing and indications for imaging that could not be accounted for in the analysis. Lastly, though the same criteria for graft retention as in the previous study were used [10], there is no standardized method for assessing graft retention, meaning that the retention rate could vary significantly if a different definition were used.

## CONCLUSION

Pedicled thymic flaps can be safely harvested using a minimally invasive approach. Postoperative CT demonstrated that most grafts remained adjacent to the bronchial stump for over a year after surgery. This technique may offer a viable option for patients at high risk of developing a delayed BPF.

## Supplementary Material

ivaf012_Supplementary_Data

## Data Availability

The data underlying this article will be shared on reasonable request to the corresponding author.

## References

[1] Asamura H , Naruke T, Tsuchiya R, Goya T, Kondo H, Suemasu K. Bronchopleural fistulas associated with lung cancer operations. Univariate and multivariate analysis of risk factors, management, and outcome. J Thorac Cardiovasc Surg 1992;104:1456–64. 1434730

[2] Ichinose J , Hashimoto K, Matsuura Y, Nakao M, Okumura S, Mun M. Risk factors for bronchopleural fistula after lobectomy for lung cancer. J Thorac Dis 2023;15:3330–8. 37426169 10.21037/jtd-22-1809 PMC10323567

[3] Wang Y , Zhu M, Pan Y, Yu K. Long-term follow up and comparison between conservative and interventional therapy in postoperative bronchopleural fistula-a cohort study. J Thorac Dis 2023;15:1210–6. 37065580 10.21037/jtd-22-1426 PMC10089856

[4] Matsunaga T , Suzuki K, Hattori A, Fukui M, Takamochi K. Risk factors for bronchopleural fistula based on surgical procedure and sex in 4794 consecutive patients undergoing anatomical pulmonary resection. Surg Today 2024;54:617–26. 37924339 10.1007/s00595-023-02761-2

[5] Steimer D , Coughlin JM, Yates E et al Empiric flap coverage for the pneumonectomy stump: how protective is it? A single-institution cohort study. J Thorac Cardiovasc Surg 2024;167:849–58. 37689236 10.1016/j.jtcvs.2023.08.050

[6] Zhao R , Guan X, Zhang P et al Development of postoperative bronchopleural fistula after neoadjuvant immunochemotherapy in non-small cell lung cancer: case reports and review of the literature. J Cancer Res Clin Oncol 2024;150:175. 38573518 10.1007/s00432-024-05683-9 PMC10995031

[7] Rami-Porta R , Nishimura KK, Giroux DJ et al; Members of the IASLC Staging and Prognostic Factors Committee and of the Advisory Boards, and Participating Institutions. The International Association for the Study of Lung Cancer Lung Cancer Staging Project: proposals for Revision of the TNM Stage Groups in the Forthcoming (Ninth) Edition of the TNM Classification for Lung Cancer. J Thorac Oncol 2024;19:1007–27. 38447919 10.1016/j.jtho.2024.02.011

[8] Li S , Fan J, Zhou J, Ren Y, Shen C, Che G. Residual disease at the bronchial stump is positively associated with the risk of bronchoplerual fistula in patients undergoing lung cancer surgery: a meta-analysis. Interact CardioVasc Thorac Surg 2016;22:327–35. 26614527 10.1093/icvts/ivv327 PMC4986554

[9] Matsuoka K , Imanishi N, Yamada T et al Clinical results of bronchial stump coverage using free pericardial fat pad. Interact CardioVasc Thorac Surg 2016;23:553–9. 27338871 10.1093/icvts/ivw193

[10] Karasaki T , Fujimori S, Suzuki S, Kikunaga S. Retention rate of free pericardial fat grafts after bronchial stump coverage. Thorac Cardiovasc Surg 2024;72:646–50. 38815589 10.1055/a-2335-9986

