# Supplementary material for: Long-term retention of the pedicled thymic flap after bronchial stump coverage
Source: Interdiscip Cardiovasc Thorac Surg. 2025 Jan 28;40(2):ivaf012. doi: 10.1093/icvts/ivaf012 (PMC11806951; doi:10.1093/icvts/ivaf012)
Supplement: ivaf012_Supplementary_Data [file ivaf012_supplementary_data.zip › ivaf012_Supplementary_Table_1.docx]

**Supplementary Table 1.** The intervals between the date of surgery and the postoperative CT scans.

| Patient number | CT (days) |
| --- | --- |
| #1 | 62[regressed] |
| #2 | 688[regressed] |
| #3 | 2, 10, 21, 34, 66, 101[regressed] |
| #4 | 45, 69, 203, 476[regressed] |
| #5 | 74, 123, 188, 203 |
| #6 | 84, 119, 121, 228, 308[regressed] |
| #7 | 179, 233 |
| #8 | 3, 264 |
| #9 | 329, 960[regressed] |
| #10 | 376, 386[regressed] |
| #11 | 35, 139, 321, 413, 583 |
| #12 | 169, 268, 321, 421, 526, 582, 588 |
| #13 | 120, 268, 330, 449, 512, 561, 652, 729, 820, 877, 933 |
| #14 | 35, 56, 63, 73, 87, 190, 255, 310, 379, 445, 582, 645, 743, 813, 883, 994 |
| #15 | 365, 1062 |
| #16 | 1538 |
