# Supplementary figures and images for: Long-term retention of the pedicled thymic flap after bronchial stump coverage
Source: Interdiscip Cardiovasc Thorac Surg. 2025 Jan 28;40(2):ivaf012. doi: 10.1093/icvts/ivaf012 (PMC11806951; doi:10.1093/icvts/ivaf012)

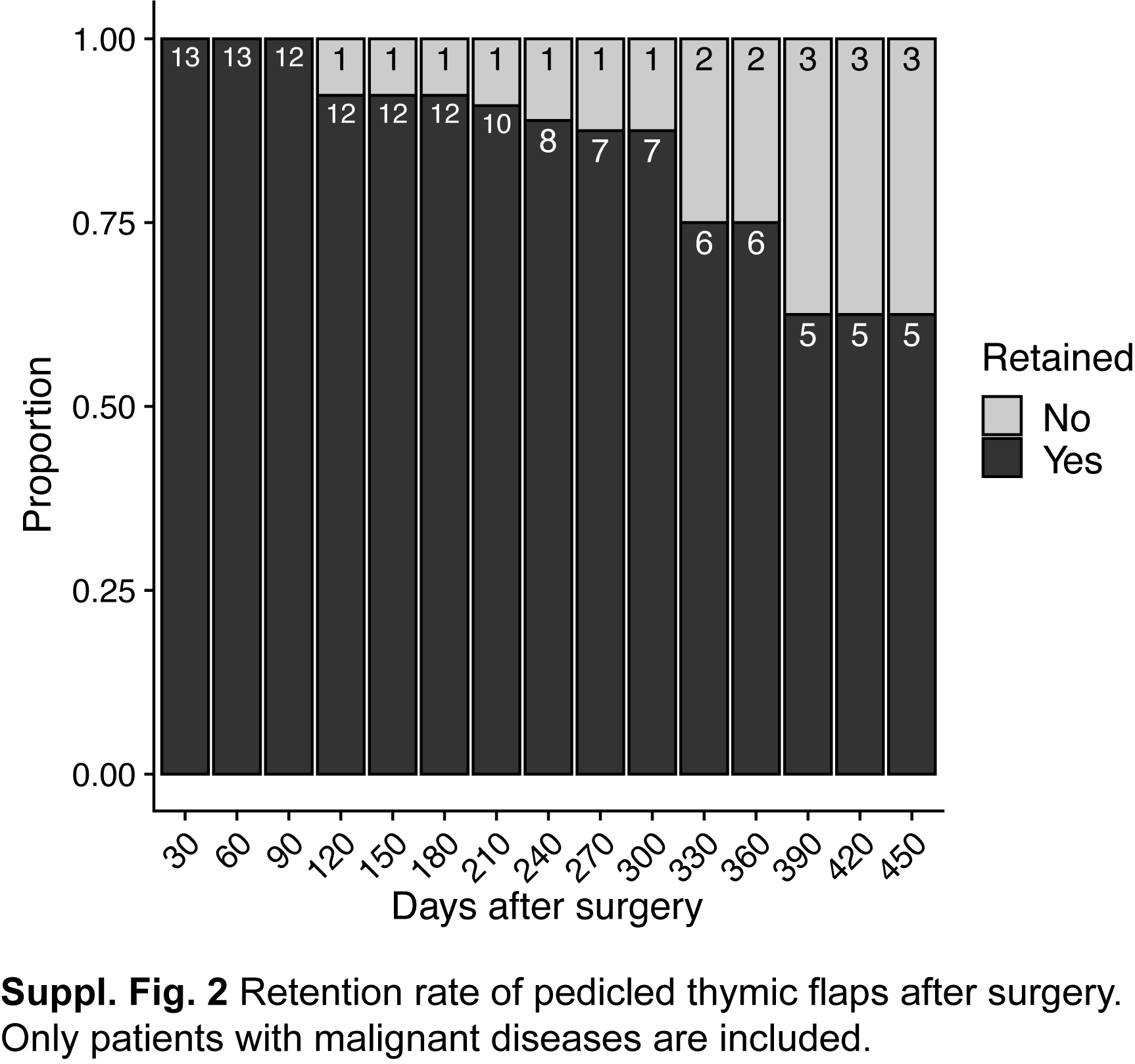

Supplement: ivaf012_Supplementary_Data [file ivaf012_supplementary_data.zip › Suppl.tif.Fig2]

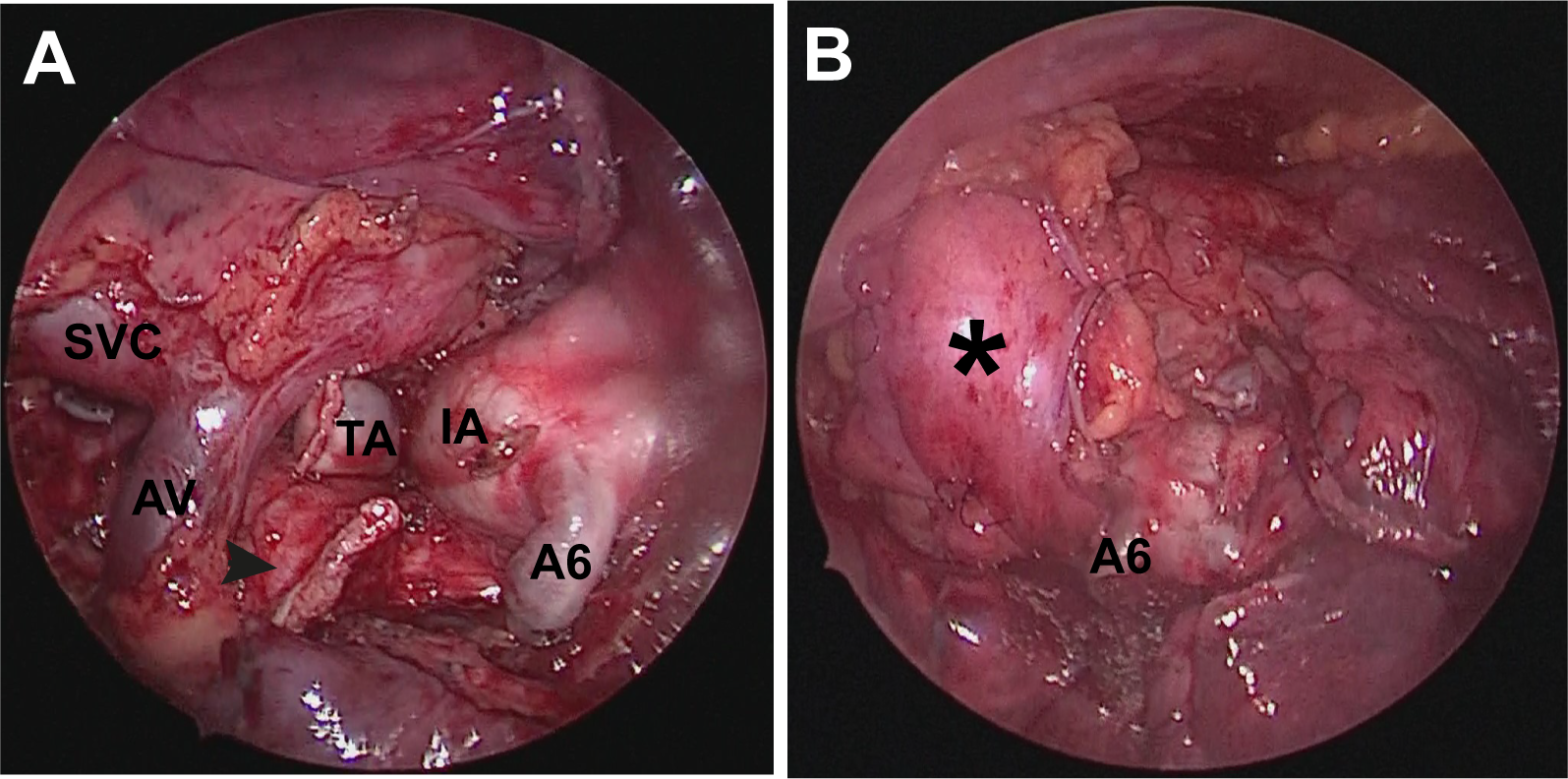

Supplement: ivaf012_Supplementary_Data [file ivaf012_supplementary_data.zip › Sup.tif.Fig1_1111]

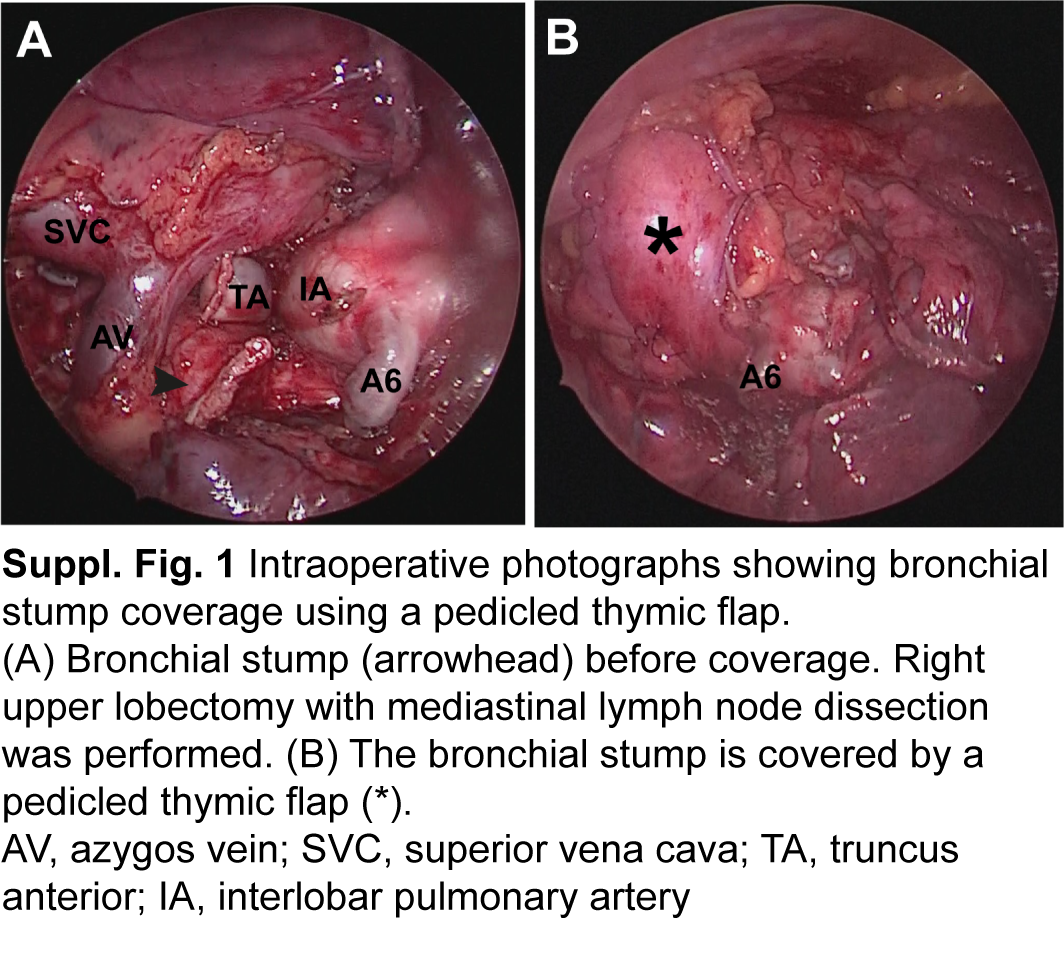

Supplement: ivaf012_Supplementary_Data [file ivaf012_supplementary_data.zip › Suppl.tif.Fig1]
